# A Post-Quantum Secure RFID Authentication Protocol Based on NTRU Encryption Algorithm

**DOI:** 10.3390/s26031038

**Published:** 2026-02-05

**Authors:** Hu Liu, Hengyu Wu, Ning Ge, Qingkuan Dong

**Affiliations:** 1 State Key Laboratory of Intelligent Vehicle Safety Technology, Chongqing Changan Automobile Co., Ltd., Chongqing 401122, China; liuhu@changan.com.cn; 2 Department of Electronic Engineering, Tsinghua University, Beijing 100084, China; gening@tsinghua.edu.cn; 3 School of Telecommunications Engineering, Xidian University, Xi’an 710071, China; qkdong@xidian.edu.cn

**Keywords:** RFID System, NTRU algorithm, quantum-resistant cryptography, mutual authentication

## Abstract

As a non-contact identification technology, RFID (Radio Frequency Identification) is widely used in various Internet of Things applications. However, RFID systems are highly vulnerable to diverse attacks due to the openness of communication links between readers and tags, leading to serious security and privacy concerns. Numerous RFID authentication protocols have been designed that employ hash functions and symmetric cryptography to secure communications. Despite these efforts, such schemes generally exhibit limitations in key management flexibility and scalability, which significantly restricts their applicability in large-scale RFID deployments. Confronted with this challenge, public key cryptography offers an effective solution. Taking into account factors such as parameter size, computational complexity, and resistance to quantum attacks, the NTRU algorithm emerges as one of the most promising choices. Since the NTRU signature algorithm is highly complex and requires large parameters, there are currently only a few NTRU encryption-based RFID authentication protocols available, all of which exhibit significant security flaws—such as supporting only one-way authentication, failing to address public key distribution, and so on. Moreover, performance evaluations of the algorithm in these contexts are often incomplete. This paper proposes a mutual authentication protocol for RFID based on the NTRU encryption algorithm to address security and privacy issues. The security of the protocol is analyzed using the BAN-logic tools and some non-formalized methods, and it is further validated through simulation with the AVISPA tool. With the parameter set (N, p, q) = (443, 3, 2048), the NTRU algorithm can provide 128 bits of post-quantum security strength. This configuration not only demonstrates greater foresight at the theoretical security level but also offers significant advantages in practical energy consumption and computation time when compared to traditional algorithms such as ECC, making it a highly competitive candidate in the field of post-quantum cryptography.

## 1. Introduction

Radio Frequency Identification (RFID) is a key technology in the perception layer of the Internet of Things, used for item identification and tracking. It enables automatic object recognition in a contactless manner. Typically, an RFID authentication system consists of one backend server, multiple readers, and a large number of electronic tags. The tag is attached to an item, and the reader activates it via an electromagnetic field generated by radio frequency signals, establishing wireless communication. Electronic tags are characterized by low cost, long lifespan, ease of use, batch identification capability, and real-time data collection. They have been widely applied in logistics tracking, smart warehousing, smart cities, medical management, and intelligent transportation, significantly improving efficiency and reducing costs. In recent years, RFID has been evolving toward higher frequency bands (such as UHF and millimeter wave), lower power consumption (e.g., passive RFID), and greater intelligence (e.g., sensor-integrated RFID). However, the large-scale deployment of RFID systems has raised concerns regarding data security and privacy protection. Due to the openness of communication links between readers and tags, RFID systems are vulnerable to various attacks, such as item tracking, tag counterfeiting, data tampering and leakage, and unauthorized access. Meanwhile, RFID systems are constrained by cost and cannot employ complex, resource-intensive security solutions. For general long-life tags, power consumption needs to be controlled within the range of 10 to 100 microwatts, which imposes certain requirements on the algorithm design. Therefore, it is necessary to design secure, efficient, and cost-controlled RFID authentication protocols to safeguard the security of RFID communications.

When constructing high-security-strength authentication protocols, it is essential to fully consider the implementation characteristics and security requirements of their target deployment environments. Some existing security mechanisms designed for minimalistic hardware platforms, such as ultra-lightweight schemes based on simple substitution-permutation, logical operations, and Physical Unclonable Functions (PUFs) [1] prioritize cost minimization in their design. These schemes typically employ mechanisms like key ratcheting to compensate for insufficient cryptographic strength, but the fundamental security functions they provide are limited, making it difficult to meet the demands of high-security scenarios with strict requirements for identity authenticity, data confidentiality, and communication non-repudiation. In contrast, another category of lightweight schemes that aim to balance implementation complexity and security strength are designed for applications with clear needs for cryptographic security assurance, such as brand anti-counterfeiting and asset tracking. The characteristics of such platforms indicate that an ideal authentication protocol should be able to achieve robust security goals through ingenious cryptographic constructions without excessive reliance on dedicated hardware resources.

Therefore, this research focuses on exploring a protocol capable of meeting such high-security and strong-authentication demands. Our core objective is not to passively adapt to a specific hardware cost threshold but to proactively design an authentication protocol that is theoretically rigorous in cryptography, complete in security attributes (such as achieving mutual authentication and resisting quantum attacks), and sufficiently streamlined and efficient in structure. This approach ensures that its security strength is inherently guaranteed by advanced cryptographic primitives (such as the NTRU algorithm), thereby making it suitable for application scenarios with higher security requirements.

Currently, there are two primary approaches to enhancing the security of RFID systems: physical methods and cryptographic methods. Physical methods require additional hardware devices and face security issues arising from external environments, such as tag destruction or disposal. Consequently, security authentication based on cryptographic protocols has gradually become a research focus. Among existing security authentication solutions for lightweight RFID tags, those based on symmetric encryption [2] or hash functions [3] have been widely studied. These schemes feature low computational complexity and minimal resource consumption, making them suitable for low-cost passive electronic tags. However, such symmetric cryptography-based designs share a common drawback: inflexible key management and poor scalability, which hinder the large-scale deployment of RFID tags. Key management thus becomes a systemic bottleneck. With the inherent advantage in flexible key management, public key cryptography effectively addresses this problem and demonstrates unique value in large-scale deployment scenarios. Nevertheless, public key mechanisms are mostly built upon operations in groups or rings, which are computationally intensive and require substantial hardware resources. As a result, they are generally unsuitable for RFID tags without significant optimization or tailored design.

In recent years, when exploring authentication technologies based on public-key cryptography, the Quadratic Residue algorithm [4] and the Elliptic Curve Cryptography (ECC) algorithm [5] have been extensively studied due to their relative advantages in parameter size and computational complexity. However, from the perspective of constructing future-oriented high-security authentication protocols, both traditional schemes suffer from fundamental limitations: their security cannot withstand quantum computing attacks. Furthermore, the inherent computational complexity of large-number modular square operations in Quadratic Residue and point multiplication operations in ECC also poses challenges for protocol designs that pursue efficiency and simplicity. Meanwhile, the XMSS algorithm [6], which is based on hash function design and has been adopted by NIST as a post-quantum signature standard, has garnered attention for its exceptional resistance to quantum attacks. However, this scheme requires the signer to strictly maintain and update the private key state index—a management requirement that conflicts with the design paradigm of many stateless, lightweight interactive authentication protocols, thereby limiting its applicability. Taking into comprehensive consideration the theoretical security strength, resistance to quantum attacks, and simplicity of protocol implementation, the NTRU encryption algorithm demonstrates significant advantages. Not only has it been listed as an important candidate in the NIST post-quantum cryptography standardization process, but more importantly, its core computational structure (such as convolution operations based on polynomial rings) inherently lends itself to constructing authentication protocols with clear computational flows and streamlined interaction rounds while achieving high security strength. This makes NTRU a highly promising cryptographic foundation for designing post-quantum authentication protocols that combine both high security and high efficiency.

As a significant representative of post-quantum cryptography, the NTRU algorithm is regarded as a cornerstone for constructing next-generation security protocols due to its computational efficiency, compact keys, and provable resistance to quantum attacks. Although digital signature schemes based on NTRU lattices are limited in streamlined authentication protocol design because of their parameter size and computational complexity, this has precisely driven research to focus on leveraging the NTRU encryption algorithm itself more purely and effectively for building security protocols. However, the currently scarce authentication protocols designed around the NTRU encryption algorithm commonly suffer from fundamental security flaws (see the analysis in Section 2), such as offering only one-way authentication, failing to properly address public key distribution, or merely using NTRU as a simple encryption channel without deep integration into the authentication logic. These design incompletenesses result in protocols that cannot provide rigorous mutual authentication and comprehensive forward security, severely restricting the application potential of the NTRU algorithm in high-security authentication systems. Despite these challenges, the increasingly imminent threat of quantum computing to traditional public-key cryptosystems, together with the trade-offs in parameter size or operational complexity of other post-quantum candidates, renders the NTRU algorithm uniquely advantageous and irreplaceable for constructing public-key authentication protocols that combine high security strength with implementation simplicity. Consequently, the systematic research and design of a bidirectional authentication scheme that fully utilizes the core security features of the NTRU encryption algorithm and exhibits rigorous, complete protocol logic is of significant theoretical value and practical urgency. Based on the NTRU encryption algorithm, this paper proposes a secure authentication protocol applicable to RFID systems.The main contributions of this paper are summarized as follows:

(1) A bidirectional authentication framework based on the NTRU encryption algorithm is systematically proposed and implemented. This framework not only achieves mutual authentication between tags and readers while ensuring a high level of security but also addresses privacy protection and resistance to tracking for the communicating entities.

(2) By integrating BAN logic, informal analysis methods, and the AVISPA simulation tool, a rigorous and comprehensive security analysis of the proposed protocol is conducted. This analysis proves the protocol’s resilience against various attacks, including replay, man-in-the-middle, and privacy theft.

(3) Through a systematic comparison between NTRU and the classical Elliptic Curve Cryptography (ECC), the practical efficiency of NTRU is quantified across three key dimensions: storage, computation, and energy consumption. More importantly, it reveals that while providing 128-bit post-quantum security, NTRU maintains significant performance advantages—its encryption speed is over 20 times faster than that of ECC, and its energy consumption is only about one three-hundredth. This strongly substantiates the feasibility and superiority of NTRU in high-security, lightweight authentication scenarios, thereby providing a solid performance basis for its practical application in post-quantum Internet of Things security.

The remainder of this paper is structured as follows. Section 2 introduces the NTRU algorithm and reviews related work. Section 3 analyzes the security requirements. In Section 4, the design of a lightweight RFID authentication protocol based on the NTRU algorithm is presented. In Section 5, the security verification and analysis of the protocol, along with simulation results, are described. Section 6 evaluates the algorithm’s performance in terms of spatial overhead, temporal overhead, and energy consumption, followed by the conclusion in Section 7.

## 2. Related Work

### 2.1. The NTRU Algorithm

In 1998, Jeffrey Hoffstein et al. proposed NTRU (Number Theory Research Unit), a lattice-based public key cryptosystem defined over polynomial rings [7]. It was later included in the IEEE P1363 standards [8] as a post-quantum secure cryptographic algorithm. A brief description of the algorithm is as follows:

First of all, the cyclic convolution operation “⊗” is defined as follows: Let $a=a_{0}+a_{1}x+\cdots +a_{N-1}x^{N-1}$ and $b=b_{0}+b_{1}x+\cdots +b_{N-1}x^{N-1}$ be two elements in the polynomial ring $Z\left[x\right]/\left(x^{N}-1\right)$. If $c=c_{0}+c_{1}x+\cdots +c_{N-1}x^{N-1}=a⊗b$, then the coefficients $c_{k}$ are computed by the formula:

$$c_{k}=\sum_{i+j\equiv k\;(\mathrm{mod}\;N)}a_{i}b_{j}=\sum_{i=0}^{N-1}a_{i}b_{(k-i)\;\mathrm{mod}\;N}.$$

The NTRU algorithm consists of four stages: parameter setup, key generation, encryption, and decryption, as detailed below:

The NTRU cryptosystem operates through four distinct phases:

(1) System Setup

Choose parameters (N,p,q) where *N* is prime, gcd(p,q)=1, and q>2p;

Define polynomial sets $L_{f},L_{g},L_{r},L_{m}$ in the ring $R=Z\left[x\right]/\left(x^{N}-1\right)$.

(2) Key Generation

Select random polynomials $f\in L_{f}$ and $g\in L_{g}$, ensuring *f* is invertible in both $Z_{p}\left[x\right]/\left(x^{N}-1\right)$ and $Z_{q}\left[x\right]/\left(x^{N}-1\right)$.

Compute inverses:

$$F_{p}\equiv f^{-1}\;\left(\mathrm{mod}\;p\right),\;F_{q}\equiv f^{-1}\;\left(\mathrm{mod}\;q\right)$$

Private Key: $\left(f,F_{p}\right)$, Public Key: $h\equiv F_{q}\cdot g\;\left(\mathrm{mod}\;q\right)$.

(3) Encryption

For a message $m\in L_{m}$, select a random blinding polynomial $r\in L_{r}$;

Compute the ciphertext:

e≡p·h·r+m(modq).

(4) Decryption

Compute

a≡f·e(modq)

Recover the plaintext:

$$m^{\prime }\equiv a\cdot F_{p}\;\left(\mathrm{mod}\;p\right)$$

where $m^{\prime }$ is the decrypted message.

As an important candidate in the NIST post-quantum cryptography standardization process, the NTRU algorithm demonstrates considerable potential as a future core cryptographic primitive through its encryption scheme, which boasts exceptional computational efficiency, flexible parameter configuration, and compact key structure, garnering extensive research in secure protocol design. In contrast, digital signature schemes based on NTRU lattices typically face inherent complexity challenges in their design. For instance, the NTRU lattice-based digital signature scheme proposed by Xie et al. in 2016, which includes identity-based ring signature and attribute-based variants, has made some progress in terms of scheme efficiency; however, the complexity of its implementation and verification remains significant [9]. Such schemes often introduce substantial parameter sizes and computational overhead in complex protocols, which, to some extent, limits their direct applicability in specific security scenarios that demand protocol simplicity and high efficiency, such as high-strength mutual authentication. Consequently, when constructing authentication protocols that simultaneously pursue high security strength and execution efficiency, the research focus naturally centers on how to systematically integrate the NTRU encryption algorithm’s superior resistance to quantum attacks and high security strength into the core security mechanisms of protocol design, rather than directly adopting its signature schemes.

In 2005, Sunar et al. compared and evaluated the performance and advantages of three cryptographic algorithms in wireless sensor networks [10], namely Rabin (based on integer factorization), NTRU, and ECC. Their study concluded that NTRU significantly reduces computational energy consumption, making it suitable for energy strictly constrained scenarios, with specific energy consumption data provided. The findings demonstrated the cost and efficiency advantages of the NTRU encryption algorithm and confirmed its feasibility for RFID applications.

In 2023, Piljoo et al. proposed an NTRU polynomial multiplication accelerator based on shared SRAM, aiming to optimize the convolution operation efficiency of NTRU—a post-quantum cryptographic algorithm—in resource-constrained environments [11]. However, this scheme fails to address the computational bottlenecks present in the complete NTRU encryption and decryption pipeline. Although the accelerator improves the speed of polynomial multiplication (convolution), the modular inversion operations, which are equally critical for the key generation phase in the NTRU algorithm, still rely on other software or hardware modules. This results in an unbalanced acceleration effect across the entire protocol flow. Such isolated optimization of a single operation struggles to systematically enhance the execution efficiency and overall performance of the complete NTRU protocol.

### 2.2. NTRU-Based Authentication Protocol

Applying the high-security-strength NTRU encryption algorithm to mutual authentication and key agreement protocols represents a significant research direction in the field of post-quantum cryptography. While multiple studies have explored this direction, most existing schemes exhibit notable deficiencies in either the completeness of protocol design or their resilience against attacks, failing to adequately meet the requirements of high-security authentication scenarios.

In 2015, Lee et al. proposed an NTRU-based anonymous authentication and key agreement scheme [12], which suffered from fundamental flaws in its protocol mechanism design. A security analysis by Raylin et al. in 2016 identified logical vulnerabilities in the zero-knowledge proof mechanism upon which the original scheme relied, rendering it incapable of resisting active attacks; an attacker could even impersonate a legitimate user without possessing any secret information [13].

In 2018, Chen et al. introduced an NTRU-based anonymous handover authentication protocol, whose core contribution lies in utilizing temporary pseudonym IDs to achieve user anonymity [14]. However, subsequent analysis revealed design flaws in the adopted anonymity mechanism, which failed to withstand targeted correlation attacks; thus, the scheme’s security did not meet its intended objectives.

In 2019, Liu et al. explored the application of a lightweight NTRU algorithm to mobile secure communication protocols [15]. While this work aimed to enhance the protocol’s resistance to quantum attacks using NTRU, the scheme itself lacked a complete formal security proof, leaving its claimed security strength without rigorous theoretical underpinning.

In 2023, Yang et al. designed a more complex anonymous certificateless authentication and key agreement protocol for Vehicular Ad Hoc Networks (VANETs) [16]. This scheme integrated the NTRU algorithm, digital signatures, and certificateless cryptography. Its nested structure of multiple cryptographic primitives, while enhancing functionality, also introduced significant complexity challenges for security boundary analysis and practical deployment.

Recently, K. B. Aneesh Kumar et al., in 2025, proposed a forward-looking framework for quantum security [17]. This framework innovatively combines NTRU post-quantum cryptography with quantum key distribution and chaotic encryption, aiming to construct a multi-layered defense system for multimedia data. Nevertheless, this "three-in-one" hybrid architecture is tailored for specific design goals, and its implementation complexity creates a pronounced mismatch with the requirements for efficient and streamlined design in general mutual authentication protocol scenarios.

In summary, existing research on NTRU-based authentication protocols either exhibits deficiencies in fundamental security mechanisms and security proofs, or suffers from design complexity and specificity of target scenarios, making them difficult to directly apply to mutual authentication contexts that impose stringent demands on both security and efficiency. Consequently, designing a secure, structurally streamlined mutual authentication protocol that fully leverages the high-security strength and quantum-attack resistance of the NTRU algorithm presents clear research value and urgency.

### 2.3. NTRU-Based Authentication Protocol for RFID Systems

Due to the stringent constraints on computational cost and resources of RFID tags, designing authentication protocols for RFID systems presents significant challenges. In response, scholars worldwide have proposed several RFID authentication protocols based on the NTRU algorithm in earlier stages.

For instance, in 2007, Selim et al. proposed a privacy-aware RFID infrastructure using public key cryptography (PKC) [18]. In this infrastructure, different readers can interrogate RFID tags for various purposes. The scheme demonstrated the feasibility of implementing the NTRU public key cryptosystem on low-power, small-area passive RFID tags (such as Class 2 tags), with experiments showing that the hardware implementation of NTRU required only about 3300 logic gates and 20 µW power consumption. The study also claimed that its performance was comparable to symmetric encryption schemes like AES, though the accuracy of this conclusion remains somewhat debatable.

In 2009, Cai et al. proposed an RFID communication security protocol using the NTRU public key cryptosystem [19]. They employed a constructed randomization function to dynamically and randomly map the plaintext in the NTRU system. However, the protocol’s authentication process contained critical flaws: it failed to achieve mutual authentication. Attackers could initiate authentication requests without any key. In practice, the tag responded unconditionally and did not actively verify any server credentials (such as signatures or keys), passively waiting for decryption results instead.

In the same year, Zhang et al. proposed a new RFID authentication protocol adopting the NTRU public key encryption algorithm [20]. In this protocol, the server authenticated the tag by verifying whether the random number “*r*” was equal to “$r_{old}$” or “$r_{new}$”. This approach had obvious vulnerabilities: attackers could intercept previous legitimate messages and replay them in subsequent sessions. Since the server only checked whether the $r^{\prime }s$ value belonged to {$r_{old}$, $r_{new}$} without verifying the freshness of the random number, replay attacks could succeed. Additionally, the protocol did not effectively bind the server-generated random number to the tag’s response, allowing attackers to mix and match messages from different sessions. The study also discussed the spatial overhead and hardware resource consumption of the protocol; however, most calculations directly adopted conclusions from prior research and could not accurately reflect the performance of the proposed scheme itself.

In 2012, Li et al. proposed a new RFID authentication protocol [21] and provided a systematic, formal description and analysis of its security by combining the security of the NTRU public key encryption system with the universal composability model. However, in this protocol, random numbers were transmitted directly in plaintext, making them susceptible to tampering or replay by attackers.

While the aforementioned schemes have explored the feasibility of applying NTRU to RFID systems, they all exhibit certain security issues, such as implementing only one-way authentication, failing to address public key distribution or merely utilizing NTRU as an encryption channel, thereby failing to translate the high-security potential of the NTRU algorithm into practical security guarantees at the protocol level. Due to the inherent characteristics of the NTRU algorithm and the substantial parameter sizes associated with NTRU signatures, research on NTRU for RFID authentication has been scarce in recent years. This paper will employ the NTRU encryption algorithm as a tool to investigate bidirectional authentication protocols for RFID tags, aiming to provide a viable solution for enhancing the security of RFID systems.

### 2.4. Chaos-Based Encryption for IoT and Multimedia Security

Beyond structured cryptographic primitives like NTRU, chaos-based encryption has emerged as a promising alternative for lightweight and high-security applications, particularly in multimedia and IoT security. Chaotic systems are characterized by sensitivity to initial conditions, ergodicity, and pseudo-randomness, properties that can be harnessed to design efficient encryption schemes. Recent surveys, such as “Chaos-Based Video Encryption Techniques: A Review” [22], systematically summarize multi-layer and multi-directional encryption concepts using chaos. These approaches often combine permutation (scrambling pixel/bit positions) and diffusion (altering pixel/bit values) phases in innovative ways, achieving robust security suitable for real-time video data. In the context of RFID and IoT, chaos-based lightweight stream ciphers or permutation networks have been explored for sensor data encryption and authentication. For instance, a lightweight chaos-based encryption scheme designed specifically for IoT-enabled systems demonstrates how dynamic chaotic sequences can secure communication while meeting stringent resource constraints [23]. While their security proofs often differ from those of number-theoretic or lattice-based schemes, the principles of leveraging complex, deterministic dynamics for confusion and diffusion offer valuable insights. Integrating such concepts, perhaps in hybrid designs alongside post-quantum primitives like NTRU for key establishment, could be a fruitful direction for building ultra-lightweight yet resilient security frameworks for future IoT deployments, an avenue we consider for future work.

## 3. RFID Framework and Security Needs

### 3.1. RFID System Fundamentals and Framework

**Figure 1:**
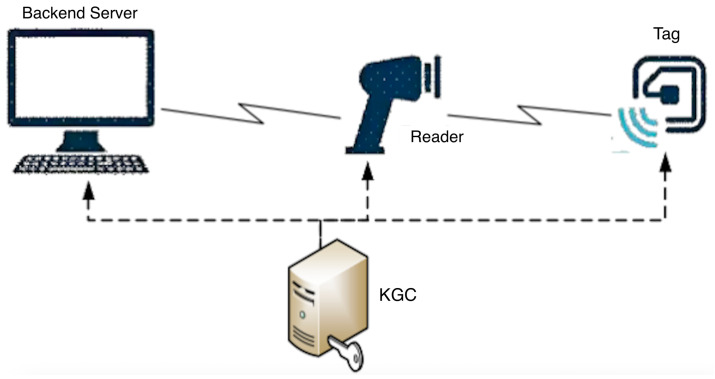
RFID System Architecture Diagram.

As shown in Figure 1, an RFID system primarily consists of three nodes—the reader, tag, and backend server—along with a Key Generation Center (KGC).

Backend Server: Serving as the core infrastructure of the RFID system, the backend server is generally assumed to be a trusted entity. It is responsible for maintaining all data related to tags/readers and item information, providing data query and storage services to readers and authorized users. The backend database maintains a critical dataset storing essential information of all electronic tags (IDs, keys, and other security data) as well as data related to the items to which they are bound. In recent studies, the use of cloud services or blockchain to replace the backend server has been discussed to enhance security and reduce operational costs.

Reader: The reader activates electronic tags via radio frequency signals and reads/writes information. It connects to the backend database through wired or wireless links. Wired connections are generally assumed to be secure channels, while wireless connections typically require establishing a secure authenticated channel based on cryptographic techniques. For example, before each connection, the reader and backend server negotiate a session key K and transmit messages using the $E_{K}\left(m||h\left(m\right)\right)$ mode. Therefore, in studying RFID system security, the channel between the reader and backend server is often assumed to be secure. In addition to facilitating communication between tags and the backend server, the reader also performs operations such as encryption, decryption, and authentication.

Tag: Electronic tags contain an internal chip and built-in antenna, providing capabilities for data storage, computation, and transmitting/receiving radio frequency signals. Each electronic tag has a unique identity, $id_{T}$. Low-cost RFID systems typically use passive lightweight tags.

KGC: The Key Generation Center is primarily responsible for managing public key system parameters, generating and maintaining keys and other related secret information for each entity.

### 3.2. RFID Security Requirements

The security requirements of RFID systems generally encompass the following aspects:

Untraceability: An attacker should not be able to track a tag by analyzing its response messages, nor trace the behavior or location of its holder through communication data.

Data Confidentiality: Sensitive information—such as the tag’s identity, cryptographic keys, data related to the item bound to the tag, and other secret information—should be accessible only to authorized parties.

Mutual Authentication: Both the reader and the tag must verify the legitimacy of each other’s identity to prevent spoofing, message tampering, replay attacks, and man-in-the-middle attacks. In many RFID authentication schemes, only one-way authentication from the reader to the tag is implemented, which is insufficient for systems with even moderate security requirements. Unauthorized readers may compromise tag privacy by extracting sensitive information from tags.

Forward Secrecy: Even if an attacker compromises the master key of a tag or reader and obtains current communication data, they should not be able to decrypt previously intercepted messages.

Backward Secrecy: If a temporary session key of a tag or reader is compromised, the attacker should not be able to use it to decrypt subsequent RFID communication data. This is particularly important in scenarios requiring protection against persistent attacks after temporary key exposure, such as long-term deployed access control systems.

Key Generation Center (KGC) Resilience: This primarily refers to protection against DoS attacks targeting the backend server launched by unauthorized users. By impersonating readers and sending a large volume of random illegal queries, attackers may exhaust server resources, rendering the system unable to serve legitimate users or even causing system crashes.

Resistance to Denial-of-Service (DoS) Attacks: It is crucial to acknowledge that the compromise or partial failure of the KGC represents a significant threat. If the KGC’s long-term master secrets or its key generation function are compromised, an adversary could potentially generate valid credentials for arbitrary readers or tags, leading to a complete breakdown of the system’s authentication guarantees.

## 4. Protocol Design

This section presents the design of a lightweight mutual RFID authentication protocol based on the NTRU encryption algorithm, addressing the security requirements of RFID systems. The protocol comprises three phases: security assumptions, system initialization, and authentication procedure.

### 4.1. Protocol Assumptions and Symbol Definitions

In the protocol design of this paper, the following assumptions are made based on practical application scenarios:

(1) The backend server is a trusted entity.

(2) A secure authenticated channel exists between the reader and the backend server, established each time the reader connects to the server.

(3) The NTRU encryption/decryption algorithm and the hash function are secure.

(4) The Key Generation Center (KGC) is a fully trusted entity in both physical and logical terms.

Table 1 lists the notations used in the protocol.

### 4.2. Protocol Initialization

Prior to protocol execution, the RFID system must complete the following initialization procedures, which are carried out by the Key Generation Center (KGC):

The KGC first configures the parameters of the NTRU cryptosystem (refer to Section 2) and selects appropriate hash functions and random number generators.

The KGC generates an identity identifier $id_{R}$ for the reader, computes $h(id_{R})$, and generates a public–private key pair $\left(PK_{R},SK_{R}\right)$. The set $\left\{id_{R},h\left(id_{R}\right),PK_{R},SK_{R}\right\}$ is then distributed to the reader via a secure channel.

The KGC generates an identity identifier $id_{T}$ for the tag, computes $h(id_{T})$ and $E_{PK_{S}}\left(h\left(id_{T}\right)\right)$ (the hash value of the tag’s identity encrypted with the server’s public key), and generates a public–private key pair $\left(PK_{T},SK_{T}\right)$. It also creates a seed $k_{0}$ for the Hash_DRBG random number generator. Assuming *n* readers are authorized to read the tag (which is also related to the tag’s capacity), the KGC generates the hash values $h(PK_{R_{n}})$ corresponding to the public keys of the n readers separately. The set is stored in the tag:

$$\left\{id_{T},h\left(id_{T}\right),E_{PK_{S}}\left(h\left(id_{T}\right)\right),SK_{T},k_{0},h\left(PK_{R_{1}}\right),\ldots ,h\left(PK_{R_{n}}\right)\right\}$$

The KGC generates a public–private key pair $\left(PK_{S},SK_{S}\right)$ for the backend server.

The backend server stores the tag’s identity identifier $id_{T}$, the hash value of its identity $h(id_{T})$, the tag’s public key $PK_{T}$, and the information Data of the item bound to the tag, i.e., the set $\left\{id_{T},h\left(id_{T}\right),PK_{T},Data\right\}$.

### 4.3. Protocol Authentication Stage

(1) Reader R→ Tag *T*: $\mathrm{Request}=[r_{R},PK_{R}]$

The reader generates a nonce $r_{R}$, and sends $r_{R}$ together with $PK_{R}$ as an authentication request to the tag *T*. The tag does not need to store the $PK_{R}$ from the request message; it only utilizes the pre-stored hash value to verify the legitimacy.

(2)Tag T→ Reader *R*: $E_{PK_{R}}(r_{R}\left\|r_{T}\right\|E_{PK_{S}}\left(h\left(id_{T}\right)\right))$

After receiving the message, the tag computes $h^{\prime }\left(PK_{R}\right)$ using the received $PK_{R}$, and sequentially checks it against its locally stored $h(PK_{R})$. If a match is found, a legitimate reader public key is identified; if no matches occur, the reader’s identity is deemed invalid and the protocol is terminated.

If the reader is authenticated, the tag generates a nonce $r_{T}$, encrypts the concatenated string $r_{R}\left\|r_{T}\right\|E_{PK_{S}}\left(h\left(id_{T}\right)\right)$ using the reader’s public key $PK_{R}$, and sends the resulting ciphertext $E_{PK_{R}}(r_{R}\left\|r_{T}\right\|E_{PK_{S}}\left(h\left(id_{T}\right)\right))$ to the reader.

(3) Reader → Backend Server: $r_{R}\left\|r_{T}\right\|E_{PK_{S}}\left(h\left(id_{T}\right)\right)$

The reader decrypts the received ciphertext using its private key $SK_{R}$, extracting $r_{R}^{\prime }$, $r_{T}^{\prime }$, and $E_{PK_{S}}\left(h\left(id_{T}\right)\right)$. If $r_{R}^{\prime }=r_{R}$, the reader stores $r_{T}$ and forwards the message $r_{R}$, $r_{T}^{\prime }$, and $E_{PK_{S}}\left(h\left(id_{T}\right)\right)$ to the backend server; otherwise, the protocol is terminated. Note that the channel between the reader and the backend server is assumed to be a secure authenticated channel as described in Section 4. The encryption ensures the confidentiality of the transmitted message, while $r_{R}$ guarantees the freshness of the message received by the reader. At this stage, the reader only possesses the ciphertext of the tag’s identity hash value.

(4) Server → Reader: $E_{PK_{T}}(r_{T}\|r_{S}\|c\|h\left(id_{T}\right))$, $r_{R}$, $r_{S}$

Upon receiving the message, the server decrypts $E_{PK_{S}}\left(h\left(id_{T}\right)\right)$ using its private key $SK_{S}$ to obtain $h(id_{T})$. It then searches the database for the tag’s identity $id_{T}$ based on $h(id_{T})$. If a match is found, the server generates random numbers *c* and $r_{S}$, retrieves the tag’s public key $PK_{T}$ from the database, and encrypts the concatenated data $r_{T}^{\prime }\left\|r_{S}^{\prime }\|c\right\|h\left(id_{T}\right)$. The server sends $r_{R}$, $r_{S}^{\prime }$, and the resulting ciphertext $E_{PK_{T}}(r_{T}^{\prime }\left\|r_{S}^{\prime }\|c\|h(id_{T}\right))$ to the reader. If no matching entry is found, the protocol is terminated. Since the channel between the reader and the backend server is secure and authenticated, the confidentiality and authenticity of the transmitted messages are preserved. Additionally, the reader can verify the freshness of the received message using $r_{R}$.

(5) Reader R→ Tag *T*: $E_{PK_{T}}(r_{T}\|r_{S}\|c\|h\left(id_{T}\right))$

The reader first verifies the freshness of the message from the server using $r_{R}$, then stores $r_{S}$ and forwards the received message $E_{PK_{T}}(r_{T}\|r_{S}\|c\|h\left(id_{T}\right))$ to the tag.

(6) Tag T→ Reader *R*: $r_{S},c$

The tag decrypts the received message using its private key $SK_{T}$, obtaining $r_{T}$, $r_{S}$, *c*, and $h(id_{T})$. By verifying $h(id_{T})$ and $r_{T}$, the tag confirms the freshness of the message and validates that the legitimate reader and backend server have performed correct decryption. Thus, the tag authenticates the reader and returns $r_{S}$ and *c* to the reader.

(7) Reader R→ Backend Server *S*: *c*

The reader verifies that the tag has correctly decrypted the message by checking the received $r_{S}$, thereby authenticating the tag. At this point, mutual authentication between the reader and the tag is completed. The reader then sends *c* back to the backend server *S*. The backend server *S* checks whether the received *c* matches the originally generated *c* to determine the success of the authentication and proceeds with subsequent management operations.

Thus, mutual authentication between the reader and the tag is successfully accomplished through the coordination of the backend server. The full authentication procedure involves four rounds of message exchange between the reader and the tag.

### 4.4. Decryption Failure Tolerance Mechanism

The protocol features a low but non-zero decryption failure probability (less than $5\times 10^{-5}$ for the selected parameters). To address this potential operational issue, a concise fault-tolerance mechanism has been designed: after each critical decryption step in the authentication process, the receiver immediately performs format and semantic validation on the decrypted plaintext. If validation fails, possible causes include malicious attacks or inherent decryption failures of the algorithm itself. In response, the protocol adopts a unified lightweight handling strategy—immediately terminating the current session and allowing the original initiator (reader or server) to automatically initiate one retry. If a retry is performed, the probability of decryption failure decreases to approximately $25\times 10^{-10}$, which is negligible. If validation still fails after the retry, the authentication process is terminated directly and flagged as an abnormal condition. This mechanism does not require introducing complex state management on resource-constrained endpoints. With only one retry, it can prevent intermittent decryption failures in the vast majority of cases and effectively distinguish between persistent attacks and transient faults, thereby enhancing stability while ensuring protocol processing efficiency.

**Figure 2:**
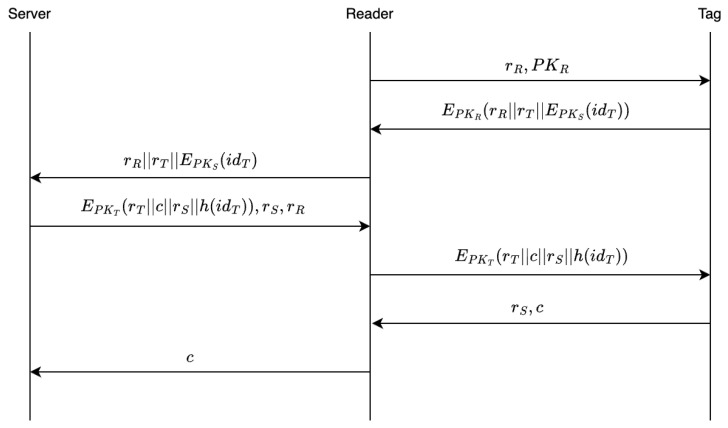
Authentication Flow of RFID Protocol.

The specific protocol flow is depicted in Figure 2:

## 5. Security Analysis, Verification and Simulation

The parameter set for the NTRU algorithm employed in this protocol is (N, p, q) = (443, 3, 2048). As shown in Table 2, against both classical and quantum attacks, this parameter set can provide a security level of 128 bits [24], which is sufficient to meet the security requirements of most practical application scenarios.

This section will conduct a security analysis of the designed protocol against the security requirements proposed in Section 3, including an informal analysis of the security functions, a BAN logic proof of the protocol, and a comparative analysis with domestic and international literature.

### 5.1. Security Analysis

(1) Anti-Tracking Capability

Tag anti-tracking capability represents a critical security consideration in RFID protocol design. This paper analyzes the anti-tracking characteristics of tags based on the established strand space model [25], with the specific methodology outlined below.

A simplified description of relevant concepts within the strand space model is provided [26]:

Let *A* denote all possible messages exchanged between entities in the protocol. The elements of *A* are termed terms, primarily comprising entity identifiers, keys, etc.

**Figure 3:**
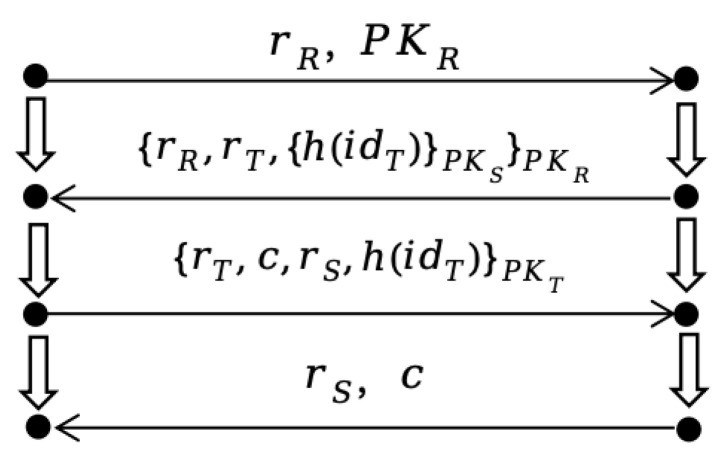
General Bundle of the Protocol.

A specific execution instance of the protocol is referred to as a bundle, denoted as *C*. The general bundle *C* for the present protocol is illustrated in Figure 3:

In a specific bundle of the RFID protocol, the strands of the reader and the tag are denoted as $s_{R}$ and $s_{T}$, respectively. The tag executing strand $s_{T}$ is denoted as *T*.

The tag strand $S_{C_{i}}=<-r_{Ri},PK_{R_{i}},-r_{Si},-c_{i},$

$$+{\left\{r_{Ri},r_{Ti},{\left\{h\left(id_{Ti}\right)\right\}}_{PK_{S}}\right\}}_{PK_{R}},+{\left\{c_{i},r_{Ti},r_{Si},h\left(id_{Ti}\right)\right\}}_{PK_{Ti}}>;$$

Let another concrete bundle be $C_{j}$, where $T\left(S_{C_{j}}\right)\neq T\left(S_{C_{i}}\right)$, and $S_{C_{j}}=<-r_{Rj},PK_{R_{j}},$

$$-r_{Sj},-c_{j},+{\left\{r_{Rj},r_{Tj},{\left\{h\left(id_{Tj}\right)\right\}}_{PK_{S}}\right\}}_{PK_{R}},+{\left\{c_{j},r_{Tj},r_{Sj},h\left(id_{Tj}\right)\right\}}_{PK_{Tj}}>;$$

(i) Observational equivalence

The attacker’s set of terms is denoted as *U*, which includes the set of terms initially known to the attacker and the set of terms generated through attacker strands. As indicated in [24], for an encrypted term, if *U* does not contain the corresponding decryption key, the attacker cannot distinguish it from a random bitstring. This property is defined as observational equivalence.

Since throughout the protocol execution, $r_{T},r_{T^{\prime }}\notin U$, the strands $s_{T}$ and $s_{T^{\prime }}$ are observationally equivalent, denoted as $s_{T}≃s_{T^{\prime }}$.

(ii) Unlinkability

If $s_{T}$ and $s_{T^{\prime }}$ are executed by the same principal, the tag strands $s_{T}$ and $s_{T^{\prime }}$ are said to be linkable, denoted as $L(s_{T},s_{T^{\prime }})$. Otherwise, they are unlinkable, denoted as $\neg L(s_{T},s_{T^{\prime }})$.

Since $r_{T}\neq r_{T^{\prime }}$, the strands $s_{T}$ and $s_{T^{\prime }}$ are unlinkable.

Let *P* be an RFID authentication protocol. If $\forall s_{T},s_{T^{\prime }}$ are tag strands, $s_{T}≃s_{T^{\prime }}$ and $\neg L(s_{T},s_{T^{\prime }})$, then *P* achieves tag untraceability [27].

From processes (i) and (ii), it follows that the tag satisfies anti-tracking capability.

(2) Tag Privacy

Tag data privacy encompasses the tag’s own identity information $id_{T}$, the tag’s private key $SK_{T}$, and the item information Data associated with the tag. Throughout the protocol execution, the tag’s identity information is never transmitted in plaintext, and even the reader cannot obtain it from the messages. The tag’s private key $SK_{T}$ is stored only within the tag, and no entity other than the tag possesses knowledge of it. It is solely used for decryption and does not need to be transmitted. The item information bound to the tag is stored and managed exclusively in the backend server.

Additionally, each time the reader initiates an authentication request, it computes a hash value based on its identity and a nonce to launch the request, which to some extent protects the identity privacy of the reader.

(3) Mutual Authentication

This protocol achieves mutual authentication between the reader and the tag using the NTRU public-key encryption algorithm. A formal verification based on BAN-logic is provided in Section 5.2, demonstrating that the protocol can resist various types of attacks, including identity spoofing, message tampering, replay attacks, and man-in-the-middle attacks.

(4) Forward/Backward Secrecy

This protocol is designed to achieve secure mutual identity authentication and entity privacy protection. Its core security objectives are distinct from establishing session keys for encrypting subsequent data transmissions. Therefore, it does not incorporate an explicit session key agreement or derivation phase. RFID tags inherently provide item identification capabilities, meaning that once a tag’s identity is recognized, both the tag and its associated item information can be retrieved from the backend database for necessary processing. Since no data transmission is required after authentication, there is also no need to negotiate a session key.

Within this context, the forward secrecy property claimed by the protocol is more precisely defined as session unlinkability and forward security of authentication credentials. Even if an adversary compromises the tag’s long-term private key $SK_{T}$ in the future and decrypts previously captured ciphertexts $E_{PK_{T}}(r_{T}\|r_{S}\|c\|h\left(id_{T}\right))$, they can only recover the session-specific, one-time random numbers $r_{T},r_{S},c$ and the hash value $h(id_{T})$. Since these random components are generated independently per session and are cryptographically unrelated to the long-term key, the adversary cannot use them to derive valid authentication credentials for other sessions or link multiple sessions to the same tag. This effectively ensures the privacy of the tag’s identity and the isolation between sessions, thereby achieving the intended forward security goal for an authentication system.

Correspondingly, as no session keys are ever established, there is no key material whose compromise could jeopardize the security of future sessions. Thus, the protocol inherently provides backward secrecy in the same context. This design aligns perfectly with the security model of typical RFID applications, where the primary concern is robust and private authentication rather than the confidentiality of a continuous data stream, allowing the protocol to avoid the overhead associated with dynamic key management in resource-constrained environments.

(5) Resistance to DoS Attacks

In the proposed authentication protocol, the backend server is susceptible to DoS attacks due to the computational and query overhead incurred when processing authentication requests. To mitigate such threats, the following countermeasures are implemented:

Attackers may impersonate readers to send random query requests to the backend server, aiming to exhaust server resources through computationally intensive operations (e.g., NTRU decryption) or excessive queries, thereby degrading service availability for legitimate users.

The Denial of Service (DoS) attack mitigation mechanism established in this protocol is a layered defense system. Its first line of defense is precisely based on the core assumption: a secure authenticated channel exists between the reader and the backend server. During the establishment of this channel, both parties have negotiated a shared key or exchanged authentication credentials. Therefore, before the server processes any query request (including those requiring decryption of $E_{PK_{S}}\left(h\left(id_{T}\right)\right)$), it can first perform a lightweight legitimacy verification of the message source, such as by validating an attached: Message Authentication Code (MAC). This operation involves minimal computational overhead yet effectively filters out flooding requests initiated by illegal, unauthenticated communication parties. Only requests that pass this verification are forwarded to the more computationally expensive NTRU decryption stage. This design achieves a balance between protocol logic and implementation cost, making it difficult for attackers to exhaust the server’s decryption resources directly by forging a large volume of requests. Consequently, it substantively enhances the system’s resilience against DoS attacks while adhering to lightweight requirements.

### 5.2. Verification Through BAN-Logic

BAN-logic is a formal logical method used for analyzing authentication protocols, primarily applied to verify the security and correctness of protocols in distributed systems, particularly in authentication and key exchange protocols. In public-key cryptosystems, the key to mutual authentication between entities lies in confirming that the counterpart possesses the corresponding private key while ensuring session freshness. Based on the protocol assumptions, since the channel between the reader and the backend server is secure, the primary focus of the security proof centers on the identity authentication between the reader and the tag.

(1) The basic terminology of BAN logic is shown in Table 3:

(2) The inference rules of BAN-logic are as follows:

Nonce Verification Rule:

$$\frac{P|\equiv \#(X),\;P|\equiv Q|∼X}{P|\equiv Q|\equiv X}$$

Jurisdiction Rule:

$$\frac{P|\equiv Q\Rightarrow X,\;P|\equiv Q|\equiv X}{P|\equiv X}$$

Freshness Rule:

$$\frac{P|\equiv \#(X)}{P|\equiv \#(X,Y)}$$

Message Interpretation Rule:

$$\frac{P|\equiv Q|∼(X,Y)}{P|\equiv Q|∼X}$$

Belief Rule 1:

$$\frac{P|\equiv X,\;P|\equiv Y}{P|\equiv (X,Y)}$$

Belief Rule 2:

$$\frac{P|\equiv (X,Y)}{P|\equiv X}$$

Belief Rule 3:

$$\frac{P|\equiv Q|\equiv (X,Y)}{P|\equiv Q|\equiv X}$$

Message Reception Rule:

$$\frac{P|\equiv Q\overset{K}{\to }P,\;P⊲{\left\{X\right\}}_{K}}{P⊲X}$$

Hash Rule:

$$\frac{P|\equiv Q∼h\left(X_{1},X_{2},\ldots ,X_{n}\right),\;P⊲X_{1},P⊲X_{2},\ldots ,P⊲X_{n}}{P|\equiv Q∼(X_{1},X_{2},\ldots ,X_{n})}$$

(3) Derivation Process

a. Protocol Description

1. $R\to T:r_{R},PK_{R}$

2. $T\to R:E_{PK_{R}}(r_{R}\left\|r_{T}\|E_{PK_{S}}\left(h\left(id_{T}\right)\right)\right)$

3. $R\to S:r_{R},r_{T},E_{PK_{S}}\left(h\left(id_{T}\right)\right)$

4. $S\to R:E_{PK_{T}}(r_{T}\left\|c\right\|r_{S}\left\|h\left(id_{T}\right)\right),r_{S},r_{R}$

5. $R\to T:E_{PK_{T}}(r_{T}\left\|c\|r_{S}\|h\left(id_{T}\right)\right)$

6. $T\to R:r_{S},c$

7. R→S:c

b. Protocol Idealization

M1: $R◃{\left\{r_{R},r_{T},{\left\{h\left(id_{T}\right)\right\}}_{PK_{S}}\right\}}_{PK_{R}},$

$\;\;{\left\{r_{T},c,r_{S},h\left(id_{T}\right)\right\}}_{PK_{T}},r_{S},r_{R},c$

M2: $S◃r_{R},r_{T},{\left\{h\left(id_{T}\right)\right\}}_{PK_{S}},c$

M3: $T◃r_{R},h\left(id_{R}\right),PK_{R},{\left\{r_{T},c,r_{S},h\left(id_{T}\right)\right\}}_{PK_{T}}$

c. Initial Assumptions

H1: $R|\equiv \#\left(r_{R}\right),\;T\left|\equiv \#\left(r_{T}\right),\;S\right|\equiv \#\left(r_{S},c\right)$

Explanation: Each entity believes that the nonces it generates are fresh.

H2: $T|\equiv \#\left(h\left(PK_{R}\right)\right),\;S|\equiv \#\left(h\left(id_{T}\right)\right)$

Explanation: As described in Section 4 (Protocol Initialization), before each authentication session, the tag pre-stores $h(PK_{R})$ and the server pre-stores $h(id_{T})$. The tag believes that the reader’s identity value $id_{R}$ is fresh, and the server believes that the tag’s identity value $id_{T}$ is fresh.

H3: $T|\equiv R|\Rightarrow h\left(id_{R}\right),\;S\left|\equiv T\right|\Rightarrow h\left(id_{T}\right)$

Explanation: The tag believes that the reader has jurisdiction over its own identity value, and the server believes that the tag has jurisdiction over its own identity value.

H4: A secure channel exists between the reader and the backend server.

H5: Each entity possesses its own private key, and the private key is known only to the entity itself.

d. Protocol Goals

$A1:S|\equiv h(id_{T})$

Explanation: The server confirms that the received tag identity is legitimate.

$A2:T\equiv R|∼\#(r_{T})$

Explanation: The tag receives a fresh nonce $r_{T}$ from the reader, indicating that the reader has successfully decrypted the ciphertext and possesses the corresponding private key $SK_{R}$, thus authenticating the reader’s identity.

$A3:R|\equiv T|∼\#(r_{S})$

Explanation: The reader receives a fresh nonce $r_{S}$ from the tag, indicating that the tag has successfully decrypted the ciphertext and possesses the corresponding private key $SK_{T}$, thus authenticating the tag’s identity.

A4:S|≡R∣∼#(c)

Explanation: The server receives a fresh nonce *c* from the reader, indicating successful authentication, and proceeds with subsequent management operations.

e. Protocol Analysis

1. Verification of A1:

From the protocol steps:

$$T\to R:E_{PK_{R}}(r_{R}\left\|r_{T}\|E_{PK_{S}}\left(h\left(id_{T}\right)\right)\right)$$

$$R\to S:r_{R},r_{T},E_{PK_{S}}\left(h\left(id_{T}\right)\right)$$

$$S⊲{\left\{h\left(id_{T}\right)\right\}}_{PK_{S}}$$

Applying the message reception rule:

$$\frac{P|\equiv Q\overset{K}{\to }P,\;P⊲{\left\{X\right\}}_{K}}{P⊲X}$$

we obtain

$$S|\equiv T|∼h(id_{T})$$

From the assumption $S|\equiv \#(h\left(id_{T}\right))$ and the nonce verification rule,

$$\frac{P|\equiv \#(X),\;P|\equiv Q|∼X}{P|\equiv Q|\equiv X}$$

we derive

$$S|\equiv T|\equiv h(id_{T})$$

Using the assumption $S|\equiv T\Rightarrow h(id_{T})$ and the jurisdiction rule:

$$\frac{P|\equiv Q\Rightarrow X,\;P|\equiv Q|\equiv X}{P|\equiv X}$$

we conclude

$$S|\equiv h(id_{T})$$

Thus, A1 is verified.

2. Verification of A2:

$$\begin{array}{cc} R\to T: & E_{PK_{T}}(r_{T}\left\|c\|r_{S}\|h\left(id_{T}\right)\right),\\ & T⊲{\left\{r_{T},c,r_{S},h\left(id_{T}\right)\right\}}_{PK_{T}} \end{array}$$

Applying the message reception rule:

$$\frac{P|\equiv Q\overset{K}{\to }P,\;P⊲{\left\{X\right\}}_{K}}{P⊲X}$$

we obtain

$$T|\equiv R|∼(r_{T},c,r_{S},h\left(id_{T}\right));$$

By the message interpretation rule:

$$\frac{P|\equiv Q|∼(X,Y)}{P|\equiv Q|∼X}$$

we obtain

$$T|\equiv R|∼r_{T};$$

From $T|\equiv \#(r_{T})$, we have $T|\equiv R|∼\#(r_{T})$.

Thus, A2 is verified.

3. Verification of A3:

(i) First, prove $R|\equiv r_{S}$:

From $S\to R:r_{S},r_{R}$, we have $R|\equiv S|∼(r_{S},r_{R})$.

From $R|\equiv \#(r_{R})$ and the freshness rule:

$$\frac{P|\equiv \#(X)}{P|\equiv \#(X,Y)}$$

we obtain

$$R|\equiv \#(r_{R},r_{S});$$

By the nonce verification rule:

$$\frac{P|\equiv \#(X),\;P|\equiv Q|∼X}{P|\equiv Q|\equiv X}$$

we derive

$$R|\equiv S|\equiv (r_{R},r_{S});$$

Using the belief rule:

$$\frac{P|\equiv Q|\equiv (X,Y)}{P|\equiv Q|\equiv X}$$

we obtain

$$R|\equiv S|\equiv r_{S};$$

From the assumption $R|\equiv S\Rightarrow r_{S}$ and the jurisdiction rule:

$$\frac{P|\equiv Q\Rightarrow X,\;P|\equiv Q|\equiv X}{P|\equiv X}$$

we conclude that

$$R|\equiv r_{S};$$

(ii) Prove $T|\equiv r_{S}$:

$$\begin{array}{cc} S\to R\to T: & E_{PK_{T}}(r_{T}\left\|c\|r_{S}\|h\left(id_{T}\right)\right),\\ & T⊲{\left\{r_{T},c,r_{S},h\left(id_{T}\right)\right\}}_{PK_{T}}, \end{array}$$

Applying the message reception rule:

$$\frac{P|\equiv Q\overset{K}{\to }P,\;P⊲{\left\{X\right\}}_{K}}{P⊲X}$$

we obtain

$$T|\equiv S|∼(r_{T},c,r_{S},h\left(id_{T}\right));$$

From $T|\equiv \#(r_{T})$ and the freshness rule:

$$\frac{P|\equiv \#(X)}{P|\equiv \#(X,Y)}$$

we obtain

$$T|\equiv \#(r_{T},c,r_{S},h\left(id_{T}\right));$$

By the nonce verification rule:

$$\frac{P|\equiv \#(X),\;P|\equiv Q|∼X}{P|\equiv Q|\equiv X}$$

we derive

$$T|\equiv S|\equiv (r_{T},c,r_{S},h\left(id_{T}\right));$$

Using the belief rule:

$$\frac{P|\equiv Q|\equiv (X,Y)}{P|\equiv Q|\equiv X}$$

we obtain

$$T|\equiv S|\equiv r_{S};$$

From the assumption $T|\equiv S\Rightarrow r_{S}$ and the jurisdiction rule:

$$\frac{P|\equiv Q\Rightarrow X,\;P|\equiv Q|\equiv X}{P|\equiv X}$$

we conclude

$$T|\equiv r_{S};$$

(iii) Prove $R|\equiv T|∼\#(r_{S})$:

From $T\to R:r_{S}$ and $R|\equiv r_{S},T|\equiv r_{S}$, we have $R|\equiv T|∼r_{S}$.

From the proof process in (i), we have $R|\equiv \#(r_{R},r_{S})$.

Therefore, $R|\equiv T|∼\#(r_{S})$.

Thus, A3 is verified.

4. Verification of A4:

From R→S:c and S∣≡c, R∣≡c,

We have S∣≡R∣∼c;

From $S|\equiv \#(r_{S},c)$, we have S∣≡R∣∼#(c).

Thus, A4 is verified.

### 5.3. Security Simulation of the Protocol

To further validate the security of the protocol, this paper employs the AVISPA tool [28] based on the SPAN-Ubuntu 10.10 platform for modular formal verification. The tool offers a formal specification language called HLPSL (High-Level Protocol Specification Language), which enables a standardized description of the protocol and its security properties, thereby determining whether any security vulnerabilities exist in the protocol. The specific steps are as follows:

First, the authentication process of the protocol is formally described using the HLPSL language. The protocol involves three roles: tag T, reader R, and trusted third party S. In this model, the tag only interacts with the reader, while the reader relies on the trusted third party to provide updated triple data in order to complete mutual authentication with the tag. It is assumed here that the communication channel between the reader and the trusted third party is secure.

The formal description of the protocol authentication process in HLPSL is as follows:

(1) The reader sends an authentication request to the tag:

$$\mathrm{State}=0\;/\;\mathrm{RCV}(\mathrm{start})\;\Rightarrow \;{\mathrm{State}}^{\prime }:=1\;/\;\mathrm{SND}\left(r_{R}\cdot PK_{R}\right)$$

(2) The tag receives the reader’s authentication request and responds as follows:

$$\begin{array}{cc} \mathrm{State}=0 & /\mathrm{RCV}(r_{R}^{\prime }\cdot PK_{R}^{\prime })\\ \Rightarrow {\mathrm{State}}^{\prime } & :=1\\ & /\mathrm{check}(h\left(PK_{R}^{\prime }\right)=h\left(PK_{R}\right))\\ & /\mathrm{witness}(T,R,\mathrm{auth}\_1,r_{R}^{\prime })\\ & /\mathrm{SND}\left(E_{PK_{R}}\left(r_{T}\cdot E_{PK_{S}}\left(h\left(id_{T}\right)\right)\right)\right) \end{array}$$

(3) The reader receives the tag’s response and sends an update request to the trusted third party:

$$\begin{array}{cc} \mathrm{State}=1 & /\mathrm{RCV}\left(E_{PK_{R}}\left(r_{T}^{\prime }\cdot E_{PK_{S}}\_hidT\right)\right)\\ \Rightarrow {\mathrm{State}}^{\prime } & :=2\\ & /\mathrm{decrypt}\left(E_{PK_{R}}\left(r_{T}^{\prime }\cdot E_{PK_{S}}\_hidT\right),SK_{R}\right)\\ & /\mathrm{check}(r_{T}^{\prime }\mathrm{is}\mathrm{fresh})\\ & /\mathrm{SND}(r_{R}\cdot r_{T}^{\prime }\cdot E_{PK_{S}}\_hidT) \end{array}$$

(4) The trusted third party receives the reader’s update request and replies as follows:

$$\begin{array}{cc} \mathrm{State}=0 & /\mathrm{RCV}(r_{R}^{\prime \prime }\cdot r_{T}^{\prime \prime }\cdot E_{PK_{S}}\_hidT^{\prime })\\ \Rightarrow {\mathrm{State}}^{\prime } & :=1\\ & /\mathrm{decrypt}(E_{PK_{S}}\_hidT^{\prime },SK_{S})\\ & /\mathrm{witness}(S,R,\mathrm{auth}\_2,r_{R}^{\prime \prime })\\ & /\mathrm{SND}\left(E_{PK_{T}}\left(r_{T}^{\prime \prime }\cdot c\cdot r_{S}\cdot h\left(id_{T}^{\prime }\right)\right)\cdot r_{S}\cdot r_{R}^{\prime \prime }\right) \end{array}$$

(5) The reader receives the trusted third party’s response and sends an authentication message to the tag:

$$\begin{array}{cc} \mathrm{State}=2 & /\mathrm{RCV}\left(E_{PK_{T}}\left(r_{T}^{\prime \prime \prime }\cdot c^{\prime }\cdot r_{S}^{\prime }\cdot h\_idT\right)\cdot r_{S}^{\prime }\cdot r_{R}^{\prime \prime \prime }\right)\\ \Rightarrow {\mathrm{State}}^{\prime } & :=3\\ & /\mathrm{check}(r_{R}^{\prime \prime \prime }=r_{R})\\ & /\mathrm{witness}(R,T,\mathrm{auth}\_3,r_{S}^{\prime })\\ & /\mathrm{SND}\left(E_{PK_{T}}\left(r_{T}^{\prime \prime \prime }\cdot c^{\prime }\cdot r_{S}^{\prime }\cdot h\_idT\right)\right) \end{array}$$

(6) The tag receives the authentication message and replies as follows:

$$\begin{array}{cc} \mathrm{State}=1 & /\mathrm{RCV}\left(E_{PK_{T}}\left(r_{T}^{\prime \prime \prime \prime }\cdot c^{\prime \prime }\cdot r_{S}^{\prime \prime }\cdot h\_idT^{\prime }\right)\right)\\ \Rightarrow {\mathrm{State}}^{\prime } & :=2\\ & /\mathrm{decrypt}\left(E_{PK_{T}}\left(r_{T}^{\prime \prime \prime \prime }\cdot c^{\prime \prime }\cdot r_{S}^{\prime \prime }\cdot h\_idT^{\prime }\right),SK_{T}\right)\\ & /\mathrm{check}(r_{T}^{\prime \prime \prime \prime }=r_{T})\\ & /\mathrm{witness}(T,R,\mathrm{auth}\_4,r_{S}^{\prime \prime })\\ & /\mathrm{SND}(r_{S}^{\prime \prime }\cdot c^{\prime \prime }) \end{array}$$

(7) The reader confirms the tag’s response and completes the protocol authentication:

$$\begin{array}{cc} \mathrm{State}=3 & /\mathrm{RCV}(r_{S}^{\prime \prime \prime }\cdot c^{\prime \prime \prime })\\ \Rightarrow {\mathrm{State}}^{\prime } & :=4\\ & /\mathrm{check}(r_{S}^{\prime \prime \prime }=r_{S})\\ & /\mathrm{witness}(R,T,\mathrm{auth}\_5,c^{\prime \prime \prime })\\ & /\mathrm{SND}(c^{\prime \prime \prime }) \end{array}$$

On the basis described above, the HLPSL statements are executed. To date, AVISPA has integrated four distinct state-of-the-art analysis techniques in its backend, namely OFMC, ATSE, SATMC, and TA4SP. Among these, OFMC performs protocol session verification based on the written IF specification while identifying language-level errors within the protocol. ATSE employs a comprehensive set of fault-elimination techniques to execute the protocol and assists OFMC in achieving secure protocol interaction. SATMC is a model checker primarily responsible for defining attacks on the generated scheme within the model. TA4SP is mainly used to define intrusion behaviors of an attacker, subsequently determining whether security vulnerabilities exist in the protocol.

**Figure 4:**
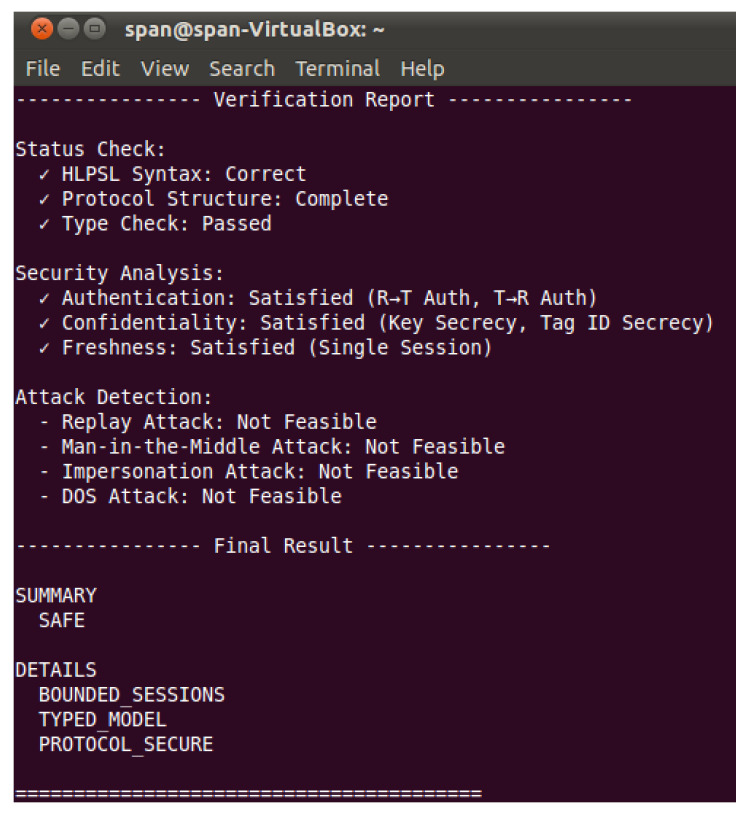
Simulation Verification Report on the Protocol.

Figure 4 shows the verification report of the protocol generated by the above analysis modules. In the formal verification based on HLPSL, the protocol passed the complete syntax, structure, and type checks. The security analysis results indicate that the protocol satisfies both authentication (including two-way authentication between the reader and the tag), confidentiality (ensuring the secrecy of the key and tag ID), and session freshness. Regarding common attack methods, the verification system confirms that replay attacks, man-in-the-middle attacks, impersonation attacks, and denial-of-service attacks are all infeasible. The final verification conclusion is that the protocol is secure, and the model satisfies the bounded session and typed security conditions. The overall assessment is “secure.”

Based on the operational mechanism of AVISPA, the security of the protocol proposed in this study has been formally validated.

### 5.4. Security Comparison Analysis

The previous sections have completed the security analysis and formal verification of the protocol. This section selects several typical protocols of the same category for comparative security analysis, with the results summarized in Table 4.

As shown in Table 4, the protocol proposed in this work addresses the limitations of existing schemes and effectively fulfills the security requirements of RFID systems.

### 5.5. Qualitative Comparison with Other Post-Quantum Schemes

To further position our NTRU-based protocol within the post-quantum cryptography landscape, we provide a qualitative comparison with other prominent NIST PQC standardization finalists and alternatives, as summarized in Table 5.

Compared to code-based or multivariate schemes, lattice-based schemes generally offer a better balance for authentication protocols. Among these, NTRU and Kyber (both based on structured lattices) are most conducive to lightweight implementations due to their small key sizes and efficient ring/module operations. While Kyber is a KEM and our protocol uses NTRU for direct encryption, the core polynomial arithmetic is comparable. FrodoKEM, based on unstructured LWE, offers conservative security but at the cost of much larger parameters, making it less suitable for RFID tags. Dilithium, as a signature scheme, inherently involves more data and computation than encryption-based authentication. Therefore, the choice of NTRU aligns well with the goal of achieving post-quantum security while maintaining feasibility for resource-constrained RFID authentication.

## 6. Performance Analysis of Algorithms in the Protocol

This section aims to conduct a systematic comparative analysis at the algorithmic level, under the same security level, between the proposed NTRU-based authentication protocol and the classical Elliptic Curve Cryptography (ECC) algorithm. The analysis will primarily focus on key performance dimensions, including core computational time, memory footprint, and theoretical power consumption characteristics. The selected benchmark algorithm is the secp256r1 curve, which serves as a typical representative of ECC algorithms and provides a classical (non-quantum) security strength of 128 bits.

### 6.1. Spatial Overhead of the Algorithm

This subsection primarily explores the storage space required for the implementation of NTRU within the protocol, which mainly includes public keys, private keys, hash values, random numbers, and ciphertexts. Among these, the storage space required for ciphertext is considered variable, as the expansion factor of ciphertext size relative to plaintext fluctuates within a dynamic range.

Public key size = $N\log_{2}q=443\times \log_{2}2048=4873\;\mathrm{bits}=610\;\mathrm{bytes}$;

Private key size = $2N\log_{2}p=2\times 443\times \log_{2}3=1404\;\mathrm{bits}=176\;\mathrm{bytes}$.

The plaintext consists of three random numbers (each 80 bits long) and a hash value (128 bits). After encryption by the NTRU algorithm, the plaintext can expand spatially by at most a factor of $\log_{p}q$. Hence, the maximum space overhead of the ciphertext is

$$\left(80\times 3+128\right)\times \log_{3}2048=2554\;\mathrm{bits}=319\;\mathrm{bytes}.$$

The comparison of spatial overhead between NTRU and ECC is presented in the following table:

As shown in Table 6, ECC demonstrates superior spatial efficiency compared to NTRU, which is fundamentally determined by the nature of their respective algorithms. The security of ECC is based on the elliptic curve discrete logarithm problem, whereas lattice-based algorithms such as NTRU require larger mathematical structures to ensure security. Furthermore, NTRU provides post-quantum security, while ECC only offers classical security. To achieve resistance against quantum attacks, NTRU incurs higher overhead costs, which entails paying a price in terms of parameter size.

To precisely fit low-cost passive tags (e.g., EPC Class 1 Gen 2) with only 1–2 KB of storage space, we implemented a key optimization in the tag’s storage design: the tag does not need to store the complete public key or the independent identity hash of any reader. Instead, it only stores a 32-byte combined hash credential $H(PK_{R})$ for each authorized reader. This credential is generated by the trusted KGC during system initialization and securely injected. During authentication, it is used to simultaneously verify the reader’s identity and the authenticity of its public key.

The cryptographic hash function used in this protocol is SHA-256, with an output length of 256 bits (32 bytes). This length provides 128-bit collision resistance security strength, which is the current industry gold standard, achieving an optimal balance between top-tier security and resource overhead. Adopting a longer, non-standard hash output (e.g., 128 bytes) would lead to excessive inflation of storage overhead, severely squeezing the space available for storing the authorization list, impairing system scalability, and offering no additional benefit to practical security levels.

Under this design, the tag’s total storage overhead can be decomposed into fixed and variable parts. The fixed overhead covers the tag’s own identity, core keys, and data required for protocol operation. The variable overhead is linearly related to the number of authorized readers with which it needs to interact.

Table 7 details the storage occupancy on the tag side:

Based on the formula Total Storage = 854 + 32n, the storage usage for typical scenarios is as follows:

Table 8 shows that even if a tag needs to interact with 10 to 30 readers, its total storage occupancy (1.15–1.77 KB) remains entirely within the typical 1–2 KB user memory capacity range of low-cost tags. This demonstrates that through careful lightweight storage optimization, this protocol achieves a post-quantum security upgrade while incurring only minimal storage cost. It effectively addresses the cost concerns associated with quantum resistance while demonstrating strong scalability, enabling it to meet the demands of large-scale RFID systems.

### 6.2. Time Overhead of the Algorithm

Time overhead is one of the most critical metrics for evaluating the performance of cryptographic algorithms. The pqm4 benchmarking project, established during the NIST post-quantum cryptography standardization process, provides an authoritative and reproducible framework for assessing algorithm performance on embedded platforms (ARM Cortex-M4) [30].

Within this framework, the NTRU algorithm with the parameter set (N, p, q) = (443, 3, 2048) requires an average of 601,800 clock cycles to complete one encryption operation (approximately 25.1 ms at 24 MHz). In contrast, the ECC (secp256r1) algorithm, which provides classic 128-bit security strength, requires an average of 23,000,000 clock cycles to complete one scalar multiplication operation (approximately 958 ms).

We prudently note that this comparative data exists within a specific context, as the performance of concrete implementations can be influenced by the degree of optimization and hardware support.

However, even considering the potential for deep optimization of ECC through methods like Montgomery multiplication, or the operation of NTRU in a basic mode that does not rely on specific hardware acceleration instructions (such as the Number Theoretic Transform, NTT), existing research and benchmark tests consistently confirm that NTRU maintains a significant leading advantage over ECC in computational speed. This is not an incidental result of specific software or hardware configurations but rather a direct manifestation of its superior algorithmic complexity in performance.

This efficiency advantage is rooted in the fundamental differences between their algorithmic kernels. The core operation of NTRU is convolution over a polynomial ring. This operation has a regular structure, is amenable to efficient software optimization, and possesses inherent parallel potential. In comparison, the core scalar multiplication of ECC is composed of a sequence of complex finite field operations (such as modular inversion and point addition), resulting in a longer computational path and difficulty in parallelization.

Therefore, for RFID systems pursuing long-term security, high throughput, or low energy consumption, choosing a post-quantum cryptographic primitive like NTRU, which possesses an efficient computational kernel, represents a more forward-looking and sustainable technological decision.

### 6.3. Energy Consumption Analysis of the Algorithm

Energy consumption is a critical metric for evaluating the feasibility of cryptographic algorithms in resource-constrained environments. Early comparative studies based on the 0.13 µm CMOS process [9] indicated significant potential for NTRU in terms of energy efficiency. However, with the advancement of semiconductor technology to 22 nm and more advanced nodes, the absolute power consumption of integrated circuits has been substantially reduced. Consequently, the specific numerical values from earlier studies are no longer suitable for direct quantitative conclusions in the current technological context.

We examine the energy efficiency trends of both algorithms through a comprehensive analysis of recent independent research. Multiple studies targeting modern embedded platforms (e.g., ARM Cortex-M4) indicate that optimized implementations of the NTRU algorithm can achieve extremely low energy consumption per operation, meeting the stringent power constraints of IoT devices. Specifically, implementations of NTRU have been shown to optimize energy consumption per operation to the microjoule (µJ) range [31].

In contrast, even with deep optimization, the classical ECC algorithm, due to the inherent computational complexity of its core operation (scalar multiplication), typically remains in the millijoule (mJ) range [32]. While providing post-quantum security, NTRU operates at an order of magnitude significantly lower in energy consumption compared to classical ECC algorithms. This fundamental energy efficiency advantage is inherently consistent with its superior computational complexity.

Therefore, we believe NTRU serves as a superior cryptographic foundation for building high-efficiency, post-quantum secure RFID systems.

## 7. Conclusions and Future Work

This paper proposes an RFID mutual authentication protocol based on the NTRU algorithm, aiming to address security and privacy issues in RFID systems. Through a systematic analysis of the protocol’s security, formal verification, and performance evaluation, the following key conclusions are drawn:

(1) High Security and Quantum Attack Resistance: The protocol is constructed based on the NTRU encryption algorithm. Its parameter set (N, p, q) = (443, 3, 2048) provides 128-bit post-quantum security strength, effectively resisting various attacks, including quantum computing. Through formal analysis using BAN logic, simulation verification with the AVISPA tool, and informal security analysis, it is confirmed that the protocol achieves mutual authentication between tags and readers and possesses critical security properties.

(2) Optimized Performance and Feasibility: In terms of performance, compared to the classical Elliptic Curve Cryptography (ECC), the NTRU algorithm demonstrates significant advantages in computational efficiency and energy consumption due to its efficient polynomial ring convolution core operation, making it more suitable for resource-constrained RFID environments. Regarding storage, by introducing a lightweight storage strategy (e.g., storing only hash credentials instead of complete public keys), the protocol successfully controls the storage overhead on the tag side within the typical capacity range (1–2 KB) of low-cost passive tags (e.g., EPC Class 1 Gen 2). It exhibits good deployment feasibility and scalability even in scenarios requiring interaction with multiple readers.

(3) Systematic Protocol Design and Analysis: This paper not only designs a complete authentication process but also incorporates a fault tolerance mechanism to address the very low probability of decryption failure inherent in the NTRU algorithm. From protocol assumptions, initialization, interaction process to security analysis, a rigorous and comprehensive design and evaluation framework has been established. Comparative security analysis also indicates that the proposed protocol outperforms existing NTRU-based RFID authentication schemes in multiple key security metrics.

In summary, the proposed protocol theoretically possesses high-strength post-quantum security and achieves efficient resource utilization in practice through performance and storage optimization. It provides a forward-looking and feasible solution for addressing authentication challenges in large-scale, high-security-demand RFID systems.

Although this study has achieved the above results, several directions warrant further exploration in future work:

(1) Adaptability in Dynamic Environments: Investigate the scalability of the protocol in multi-node, collaborative IoT scenarios, such as group authentication, and its efficiency and security in dynamic network topologies.

(2) Diversification of Backend Server Architectures: Explore upgrade paths for backend server technology, such as adopting cloud-based or blockchain-based server architectures, to further enhance system security, reliability, and scalability.

(3) Exploration of Integration with Other Post-Quantum Primitives: Investigate the possibility of hybrid designs combining NTRU with other lightweight cryptographic techniques (e.g., chaos-based encryption) to achieve a better balance between security, efficiency, and cost in specific scenarios.

(4) Enhancement of Key Management Infrastructure Resilience: Explore robust mechanisms for Key Generation Center (KGC) security, such as distributed key management based on threshold cryptography, integration with hardware security modules (HSMs), and dynamic key update protocols, to mitigate systemic risks arising from KGC compromise or failure in large-scale deployments.

## Figures and Tables

**Table 1:** Notations and Descriptions.

Notations	Description
$id_{T}$	Tag identity
$id_{R}$	Reader identity
$\left(PK_{T},SK_{T}\right)$	Tag’s Key Pair
$\left(PK_{R},SK_{R}\right)$	Reader’s Key Pair
$\left(PK_{S},SK_{S}\right)$	Backend Server’s Key Pair
$r_{X}$	Nonce generated by X
E(.)	NTRU Encryption Function
D(.)	NTRU Decryption Function
h(.)	Hash function
‖	String Concatenator

**Table 2:** Post-Quantum Parameter Sets and Security Estimates.

Classical Sec. Est.	Quantum Sec. Est.	*N*	*q*	$\left(d_{1},d_{2},d_{3},d_{g},d_{m}\right)$	Hybrid Dim	Attack β	Params Rounds	*K*	Cost	Prod. Form Search Cost	$\log_{2}$ Fail Prob.
128	128	443	2048	(9, 8, 5, 148, 115)	575	222	11	177	133	147	−196
192	128	587	2048	(10, 10, 8, 196, 157)	723	311	9	258	197	193	−139
256	128	743	2048	(11, 11, 15, 247, 204)	880	407	8	350	272	256	−112

**Table 3:** BAN-Logic Notation and Meanings.

Basic Formula	Meaning
P∼X	*P* has sent a message containing *X*
P|≡X	*P* believes message *X*
P◃X	*P* has received a message containing *X*
P⇒X	*P* has jurisdiction over *X*
#(X)	*X* is a fresh nonce
${\left\{X\right\}}_{PK}$	Ciphertext of *X* encrypted with public key PK
*K*	*K* is *P*’s public key, and except for *P* and entities it believes, no other entity knows the corresponding private key $K^{-1}$
K→P	*K* is *P*’s public key

**Table 4:** Security Performance Comparison of Different Schemes.

Scheme	Tracking Attack	Mutual Auth	Spoofing Attack	Man-in- the-Middle	Replay Attack	DoS Attack
Selim [18]	Y	Y	Y	Y	Y	N
Cai [19]	Y	N	Y	N	Y	N
Zhang [20]	Y	N	N	Y	N	Y
Li [21]	Y	N	Y	Y	Y	Y
Liu [15]	Y	Y	Y	Y	Y	N
This Work	Y	Y	Y	Y	Y	Y

Y indicates that the protocol possesses this security feature; N indicates that the protocol does not possess this security feature.

**Table 5:** Comparison of Post-Quantum Cryptography Schemes.

Scheme (Type)	Security Basis	Main Operations	Suitability for Lightweight Auth.
NTRU (this work)	Lattice (SVP)	Polynomial Multiplication (Ring)	High: Compact operations, good for constrained authentication.
Kyber (KEM)	Lattice (MLWE)	Polynomial Multiplication (Module)	Medium–High: Similar operations to NTRU, slightly larger sizes.
FrodoKEM (KEM)	Lattice (LWE)	Matrix–Vector Multiplication	Low: Large parameters, high memory/bandwidth demand.
Dilithium (Signature)	Lattice (SIS)	Polynomial Multiplication, Rejection Sampling	Medium: Designed for signing; heavier than encryption for authentication.
ECC (secp256r1)	Elliptic Curve DLP	Point Multiplication	High (Classical only): Very compact but not post-quantum secure.

**Table 6:** Comparison of Spatial Overhead between NTRU and ECC.

	NTRU (N,p,q)=(443,3,2048)	ECC (secp256r1 Curve) [29]
Public Key	610 bytes	128 bytes
Private Key	176 bytes	32 bytes
Ciphertext	319 bytes	64 bytes

**Table 7:** Tag Storage Overhead Statistics.

Storage Item Category	Specific Content	Size (Bytes)
Fixed Overhead		
	Tag Identity Identifier	16
	Hash Value of Tag Identity (SHA-256)	32
	Encrypted Hash Value of Tag Identity	614
	Tag Private Key	176
	Random Number Seed for Deterministic Random Bit Generator	16
Total Fixed Overhead		854
Variable Overhead (per item)	Reader Verification Credential (Hash of Public Key concatenated with Identity)	32
Total (Formula)		854 + 32 × n

**Table 8:** Feasibility Verification of Storage Overhead.

Number of Authorized Readers (*n*)	Total Storage (Bytes)	Approx.
10	854+32×10= 1174	1.15 KB
15	854+32×15= 1334	1.30 KB
30	854+32×30= 1814	1.77 KB

## Data Availability

No new data were created or analyzed in this study.

## Abbreviations

The following abbreviations are used in this manuscript:

NTRU	Number Theory Research Unit
ECC	Elliptic Curve Cryptography
RFID	Radio Frequency Identification
BAN	Burrows–Abadi–Needham
